# Association between Carbohydrate Intake and the Prevalence of Metabolic Syndrome in Korean Women

**DOI:** 10.3390/nu13093098

**Published:** 2021-09-03

**Authors:** Young-Ae Cho, Jeong-Hwa Choi

**Affiliations:** Department of Food Science and Nutrition, Keimyung University, Daegu 42601, Korea; youngcho914@gmail.com

**Keywords:** carbohydrate, metabolic syndrome, women, obesity, dairy, C-reactive protein

## Abstract

Carbohydrates consist of a large proportion of calories in the Asian diet. Therefore, we aimed to investigate the association between carbohydrate intake and metabolic syndrome in Korean women. A cross-sectional analysis was conducted with a total of 4294 Korean women aged 40–69 years from the Korean Genomic and Epidemiology Study (KoGES). Carbohydrate intake was calculated based on a validated food frequency questionnaire. Metabolic syndrome was defined by using the National Cholesterol Education Program, Adult Treatment Panel III (NCEPIII). Logistic regression was used to estimate the association of carbohydrate intake with metabolic syndrome and its components. In this study, high carbohydrate intake seemed to be associated with low socioeconomic status and an imbalanced diet. After adjusting for confounding factors, subjects with higher carbohydrate intake showed an increased risk of metabolic syndrome (odds ratio (OR) 1.34, 95% confidence interval (CI) 1.08–1.66, *p*-trend = 0.004, highest vs. lowest quartile [≥75.2 vs. <67.0% of energy]), particularly elevated waist circumference. This association was stronger among those with low levels of C-reactive protein (CRP) and those with low dairy intake. In conclusion, higher carbohydrate intake is associated with a higher risk of metabolic syndrome, particularly abdominal obesity, in Korean women. This association may differ according to individuals’ CRP level and dairy intake.

## 1. Introduction

Metabolic syndrome is a cluster of interrelated abnormalities, including abdominal obesity, insulin resistance, dysglycemia, hypertension, and dyslipidemia [1]. Metabolic syndrome has become an important public health concern because of its association with cardiovascular disease and type 2 diabetes [2]. Low-grade systemic inflammation is also characteristic of metabolic syndrome. Increased levels of C-reactive protein (CRP) have been reported to be associated with insulin resistance, adiposity, and other features of metabolic syndrome [3,4].

It is difficult to directly compare the prevalence of metabolic syndrome in different studies because of differences in criteria used to define metabolic syndrome, study designs, and sample size [2]. Many dietary factors have been suggested to be associated with metabolic syndrome [5,6]. It is difficult to explain the prevalence of metabolic syndrome according to single nutrients or foods. However, Asians traditionally consume a lot of rice as a staple food, thus obtaining a large proportion of calories from carbohydrates. Therefore, carbohydrate intake could play an important role in metabolic abnormalities in this population. Several studies have investigated the association between carbohydrate intake and the risk of metabolic syndrome, and they have reported mixed findings [7,8,9,10]. Some of these studies reported a stronger association in the Asian population compared to the non-Asian population [7,8], suggesting that this may be attributed to different amounts of carbohydrate consumption. In a study by Ha et al., the proportion of energy from carbohydrate was 80–82% in the highest quintile among the Korean adults, compared with 64–65% in the US adults [7]. In addition, higher proportion of carbohydrate could result in imbalanced diet. Furthermore, socioeconomic status (SES) has been suggested to contribute to health status through the intake of macronutrients. Sakurai et al. reported that older age and some aspects of SES (e.g., income and education levels) were associated with a high carbohydrate/low fat intake [11]. Therefore, SES should be considered to investigate the role of diet in metabolic syndrome.

The prevalence of metabolic syndrome increases with age, but it has different patterns according to sex. The prevalence in women is lower but catches up to that in men after the age of 60 years [2]. Because men and women have different dietary habits, work-related activities, and socioeconomic status, it is appropriate to study the role of dietary habits separately [2,12]. In addition, some studies have demonstrated stronger associations between dietary carbohydrate intake and metabolic disease in women than in men [10,13].

Based on this information, we aimed to investigate the association of carbohydrate intake with the prevalence of metabolic syndrome and its individual components in Korean women. We also investigated whether socioeconomic status affected carbohydrate intake and whether the association of carbohydrate intake with metabolic syndrome differed according to participants’ CRP level and other food group intakes that were consumed as side dishes or snacks.

## 2. Materials and Methods

### 2.1. Study Population

Korean Genomic and Epidemiology Study (KoGES) is a prospective cohort study that investigated the environmental and genetic factors affecting prevalent chronic diseases in the Korean population. As part of the KoGES, two community-based cohort study started in 2001 and since then, conducted biennial follow-up studies. Subjects aged 40–60 years were randomly selected from residents of two communities: Ansung (a rural community) or Ansan (an urban community). A total of 10,030 participants were initially enrolled and 8840 participants (4182 men and 4658 women) completed baseline survey, anthropometric and biochemical measurements, and genotyping. We used this baseline data obtained from 2001 to 2002. Detailed information on the study procedure is described elsewhere [14,15]. Of the 4658 women participants, participants were excluded due to an incomplete food frequency questionnaire (FFQ) (*n* = 164), unreliable energy intake of ≤500 kcal or >5000 kcal (*n* = 51), and missing information regarding metabolic syndrome criteria (*n* = 149). Finally, a total of 4294 women were included in this analysis.

### 2.2. Data Collection 

Information regarding the demographic and lifestyle factors (e.g., age, sex, alcohol consumption, tobacco smoking, marital status, education level, and physical activity level) of the study population was obtained by trained interviewers using a questionnaire [14]. Subjects’ physical activity levels were assessed in metabolic equivalents (METs) computed as the sum of METs for five levels of activity [16].

Dietary intake was assessed by using a validated semi-quantitative FFQ with 103 items, which was developed and validated among Koreans [17]. Each subject provided their average frequencies of consumption and typical portion sizes based on the year preceding the interview. Study participants were asked to report the frequency of their consumption of each food based on nine response options (never or barely, 1 time/month, 2 to 3 times/month, 1 to 2 times/week, 3 to 4 times/week, 5 to 6 times/week, 1 time/day, 2 times/day, or ≥3 times/day) and three portion sizes (small, medium or large). To estimate the intake of seasonal foods (i.e., fruits), participants were also asked to record the period of consumption (3, 6, or 9 months or a year). Nutritional intake was estimated by using the Food Composition Table that was developed by the Korean Health and Industry of Development Institute (7th edition).

Anthropometry and metabolic parameters were measured by trained medical staff, with subjects wearing light clothes. Body mass index (BMI) was calculated as weight (kg) divided by the square of the height (m^2^). Waist circumference was measured three times. Blood pressure was measured at least twice under comfortable conditions. Blood samples were obtained after participants had fasted for 8 h and were analyzed for biochemical markers, such as high-density lipoprotein (HDL) cholesterol, triglycerides, fasting blood glucose, and CRP.

### 2.3. Diagnostic Criteria for Metabolic Syndrome

This study defined metabolic syndrome using the updated National Cholesterol Education Program Adult Treatment Panel III (NCEP-ATPIII) (NCEP-ATPIII, 2002). To define abdominal obesity, a modified waist circumference cutoff was used [18]. Subjects were diagnosed with metabolic syndrome if they had three or more of the following: (a) central obesity (defined as waist circumference) ≥ 85 cm in women; (b) blood pressure ≥ 130/85 mmHg or use of antihypertensive medication; (c) triglyceride ≥ 150 mg/dL; (d) fasting glucose ≥ 100 mg/dL or use of antidiabetic medication; and (e) HDL cholesterol < 50 mg/dL in women. 

### 2.4. Statistical Analysis

The levels of dietary carbohydrate intake were categorized into quartiles (Qs) based on the distribution of the metabolic syndrome-free participants (Q1: <67.0, Q2: 67.0–71.4, Q3: 71.4–75.2, Q4: ≥75.2% of energy) because the presence of metabolic syndrome could change participants’ dietary habits including the amount of carbohydrate consumed. 

General characteristics of the study population were examined by metabolic syndrome status. Data are expressed as the mean ± standard deviation for continuous variables and frequency and percentage for categorical variables. Logistic regression analysis was performed to estimate the odds ratios (ORs) and 95% confidence intervals (CIs) of the risk of metabolic syndrome (or its individual components) according to the quartiles of carbohydrate intake, taking the lowest quartile group as the reference group. To select which confounders would be controlled, a backward elimination strategy was used [19]. The characteristics of the participants were categorized as follows: education level (≤middle school, high school, and ≥college), marital status (yes/no), residential location (urban area/rural area), job (professional/office worker, service/sales, agriculture/manufacturing, housewife/other), household income (low, medium, and high), alcohol intake (never/ever), smoking status (never/ever), BMI (<23, 23–25, ≥25 kg/m^2^) and physical activity (Q1: <720, Q2: 720–1155 Q3: 1155–1965, Q4: ≥1965 METs/d). Finally, a multivariable model was adjusted for age, residential area, and education level. A test for trend across quartile categories was conducted by including the median intake of each quartile as a continuous variable in the logistic regression model.

To identify factors relating carbohydrate consumption, general characteristics and the distributions of certain food and nutrient intakes among metabolic syndrome-free participants were examined across quartiles of carbohydrate intake. The significant differences for several variables across levels of carbohydrate intake were examined using the generalized linear model. Food and nutritional intake data were adjusted for total caloric intake using Willet’s residual method and were then included in the analyses [20].

To test whether these associations were affected by individuals’ inflammatory status, we conducted stratified analyses by CRP concentration. The subjects were categorized into two groups (low/high, cutoff point: 1 mg/dL). Additionally, we investigated whether intake of other food groups may affect the risk of metabolic syndrome. The subjects were categorized into two groups (low/high) based on the median intake levels of carbohydrates and other food groups in the metabolic syndrome-free participants, respectively. Then, stratified analyses were carried out according to the intake of other food groups, including fruits, kimchi (traditional fermented cabbage product), vegetables other than kimchi, vegetables, dairy foods (milk and any foods made from milk), and meats: these food groups were selected based on the amount of daily intake.

All statistical analyses were performed by using SAS 9.2 software (SAS Institute Inc., Cary, NC, USA). A two-sided *p*-value of less than 0.05 was considered statistically significant.

## 3. Results

### 3.1. Association of Carbohydrate Intake with Metabolic Syndrome and Its Components

**Figure 1:**
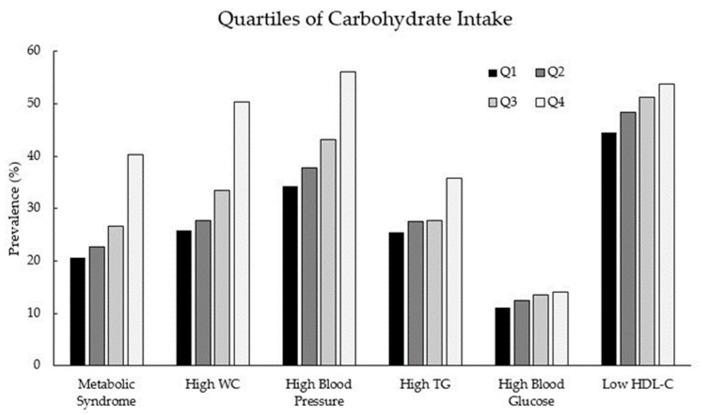
Prevalence of metabolic syndrome and its components according to the quartiles of carbohydrate intake. Q, quartile; TG, triglycerides; HDL-C, high-density lipoprotein/cholesterol; WC, waist circumference.

The mean age of the subjects in the current study was 52.3 years. A total of 1221 cases of metabolic syndrome were identified, and the prevalence of metabolic syndrome was 28.4% in this population. The participants’ characteristics according to metabolic syndrome status are presented in Supplementary Materials Table S1. Compared to those without metabolic syndrome, those with metabolic syndrome are more likely to be older (*p* < 0.001), have a higher BMI (*p* < 0.001), live in rural areas (*p* < 0.001), and be unmarried (*p* < 0.001). They were more likely to have jobs that required physical labor and were less professional (*p* < 0.001), had lower education levels (*p* < 0.001), and had lower household income (*p* < 0.001). Those in this category were less likely to drink alcohol (*p* < 0.001) and more likely to smoke tobacco (*p* = 0.018). Figure 1 shows the prevalence of metabolic syndrome and its components according to the quartiles of carbohydrate intake. Carbohydrate intake was positively associated with the prevalence of metabolic syndrome and its components. Low HDL cholesterol was the most common component of metabolic syndrome, followed by high blood pressure. 

Table 1 shows the association of carbohydrate intake with the risk of metabolic syndrome and its components. A higher carbohydrate intake was associated with an increased risk of metabolic syndrome after adjusting for covariates (OR 1.34, 95% CI 1.08–1.66, highest vs. lowest quartile, *p*-trend = 0.004). Among the components of metabolic syndrome, this association was observed for waist circumference (OR 1.32, 95% CI 1.08–1.62, highest vs. lowest quartile, *p*-trend = 0.007) and HDL cholesterol (OR 1.19, 95% CI 0.99–1.43, highest vs. lowest quartile, *p*-trend = 0.040). There was no significant association with other metabolic components.

### 3.2. Carbohydrate Intake, Socioeconomic Status, and Diet Quality 

The participants’ characteristics according to the quartiles of carbohydrate intake are presented in Table 2. Compared to those in the lowest quartile of carbohydrate intake, those in the highest quartile were more likely to be older (*p* < 0.001), live in rural areas (*p* < 0.001), and not be married (*p* = 0.001). They were more likely to have jobs that required physical labor and were less professional (*p* < 0.001), had lower education levels (*p* < 0.001), and had lower household income (*p* < 0.001). Those in this category were less likely to drink alcohol (*p* < 0.001). In terms of physical activity, carbohydrate intake was inversely associated with physical activity in the first to third physical activity groups. However, those in the highest quartiles of physical activity consumed more carbohydrates (*p* < 0.001). No differences were observed in BMI and smoking status.

The daily intakes of all macronutrients, except energy and vitamin C, were significantly lower in the highest carbohydrate group than in the lowest carbohydrate group (Supplementary Materials Table S2). The percentage of energy intake obtained from fat and protein was significantly lower in the group with higher carbohydrate intake. In addition, we examined the distribution of the intakes of other foods across the quartiles of carbohydrate intake (Supplementary Materials Table S3). Most of the intakes of the examined food groups decreased across the lowest to highest quartiles of carbohydrate intake. The intake of grains, kimchi, and fruit/fruit juice tended to be higher in the highest quartiles than in the lowest quartiles (*p* for all < 0.001). 

### 3.3. Association between Carbohydrate Intake and Metabolic Syndrome According to CRP Level and Other Food Intake

When the data were stratified by individuals’ CRP level, the association between carbohydrate intake and metabolic syndrome was significant only among those with a low level of CRP (OR 1.84, 95% CI 1.21–2.80, highest vs. lowest quartile, *p*-trend = 0.003) (Table 3). Among metabolic components, this association was observed only for elevated waist circumference (OR 1.76, 95% CI 1.24–2.51, highest vs. lowest quartile, *p*-trend = 0.002), high triglycerides (OR 1.53, 95% CI 1.03–2.26, highest vs. lowest quartile, *p*-trend = 0.026), and low HDL cholesterol (OR 1.57, 95% CI 1.15–2.14, highest vs. lowest quartile, *p*-trend = 0.001). However, none of the associations was significant among those in the high-CRP group.

Furthermore, we investigated whether the intake of other food groups (kimchi, fruits, vegetables without kimchi, vegetables, dairy foods, and meat) may affect the prevalence of metabolic syndrome. When the data were stratified by the amount of intake of the other food groups, those with high dairy intake showed a reduced risk of metabolic syndrome in the group of participants with high carbohydrate intake (OR 0.78, 95% CI 0.64–0.95, *p* = 0.014, high vs. low dairy intake) (Supplementary Materials Table S4). Among the metabolic components, this association was observed only for elevated waist circumference (OR 0.72, 95% CI 0.59–0.87, *p* < 0.001), high triglycerides (OR 0.72, 95% CI 0.59–0.87, *p* = 0.001), and low HDL cholesterol (OR 0.80, 95% CI 0.67–0.96, *p* = 0.015) (Table 4).

## 4. Discussion

In the present study, carbohydrate intake was associated with metabolic syndrome, and this association may differ according to individuals’ CRP levels and dairy food intake. Those with high carbohydrate level were more likely to be in low socioeconomic status, and their diet lacked variety and balance. 

### 4.1. Carbohydrate Intake and Metabolic Syndrome

Previous studies have reported mixed findings regarding the association between carbohydrate intake and the risk of metabolic syndrome [7,8,9,10]. The results from different countries are hard to compare because of differences in the criteria used to define metabolic syndrome, study design, and many other factors. The present study showed that high carbohydrate intake was associated with an increased risk of metabolic syndrome. Among the five components of metabolic syndrome, abdominal obesity showed the strongest association with carbohydrate intake. Abdominal obesity indicates the existence of adipose tissue dysfunction and is independently associated with hyperlipidemia (low HDL cholesterol and high triglycerides), increased insulin resistance, elevated risk of diabetes, and subclinical atherosclerosis. Therefore, identifying the determinants of abdominal obesity may reveal strategies to prevent these metabolic abnormalities [21]. 

Asians are quite prone to visceral fat accumulation, which may explain their greater tendency to develop metabolic complications of obesity at relatively low BMI values [22,23]. Some studies have reported the association of carbohydrate intake with abdominal obesity [24]. In a randomized controlled study of obese subjects with type 2 diabetes mellitus, a greater decrease in visceral fat was observed in the low-carbohydrate group than in the high-carbohydrate group. These results imply that, among isocaloric diets, a low carbohydrate diet might be more effective in reducing visceral fat, improving insulin sensitivity, and increasing HDL cholesterol levels than a high carbohydrate diet in obese subjects with type 2 diabetes mellitus [24]. Based on this evidence, higher carbohydrate intake and a higher tendency of accumulating visceral fat among Asians may increase their risk of metabolic syndrome.

### 4.2. Carbohydrate Intake and Socioeconomic Status

The proportion of carbohydrates consumed seems to be associated with socioeconomic characteristics [11]. In the current study, those with a higher carbohydrate intake were more likely to be older, have a lower level of education and household income, and live in rural areas than those with a lower carbohydrate intake. Accordingly, the association of carbohydrate intake with metabolic syndrome prevalence was attenuated after multivariable adjustment of these factors [21]. Low socioeconomic status is possibly linked to a high carbohydrate intake and a higher prevalence of metabolic syndrome [25]. It has been suggested that socioeconomic status may affect the health status of individuals due to its effect on macronutrient balance [11]. Analyses that have considered education, occupation, income, and employment status have shown that education is usually the strongest determinant of socioeconomic differences [26]. Higher levels of education may increase the ability of individuals to understand health-related information and develop health-promoting behaviors. Additionally, older age was strongly associated with high carbohydrate intake, possibly because food preferences and socioeconomic status (e.g., household income and occupation type) change with age. Finally, rural residents had a higher level of carbohydrate intake than urban residents. Generally, rural residents are older and have low socioeconomic status [27]. Based on these findings, strategies to improve diet quality need to consider socioeconomic status. 

### 4.3. Carbohydrate Intake and Diet Quality

Carbohydrate intake may play an important role in diet quality in this population because the proportion of carbohydrates was more than 70% of the total calories. Therefore, we examined differences in food and nutrient intake across quartiles of carbohydrate intake. Individuals with a high intake of carbohydrates had a higher intake of grains, but the intakes of other foods, even carbohydrate-rich foods, except for kimchi and fruit/fruit juice, were lower than those in the lower-carbohydrate groups. Lower intake of side dishes and higher intake of rice may lead to an imbalanced intake of macronutrients and other nutrients [11]. As a result, their diets lack protein, fat, and other essential minerals and vitamins. The diet of those with higher carbohydrate levels lacked variety and balance and was thus poor in regard to diet quality. Even though both the quality and quantity of carbohydrates are important, reducing the amount of carbohydrates (especially refined carbohydrates) is more effective in lowering glycemic load than reducing the overall dietary glycemic index alone [6]. High carbohydrate intake is a major characteristic of the Korean diet because rice is a staple food among Koreans. Therefore, exploring foods that are optimal alternatives for white rice to reduce metabolic syndrome incidence could be an important focus.

Therefore, other food choices, such as side dishes and snacks, may help to reduce the risk of metabolic syndrome. To examine the differences in metabolic syndrome prevalence by the amount of consumption of other food groups, we conducted stratified analyses. We found that those with higher dairy intake had a reduced risk of metabolic syndrome in the high-carbohydrate group. Because dairy foods (e.g., milk, yogurt, and cheese) are usually not included in the traditional Korean diet, their intake is lower than that in Western countries [28]. Many studies have also reported an inverse association between dairy intake and the risk of metabolic syndrome [29]. Various nutrients in milk (e.g., calcium and dairy protein) may synergistically protect against metabolic syndrome and its individual components. Calcium may increase the binding of fatty acids and bile acids in the intestine, thus increasing fecal fat excretion and inhibiting fat reabsorption [30]. Additionally, calcium may regulate body fat deposition by affecting adipocyte intracellular calcium concentrations and reducing fatty acid synthesis while increasing lipolysis and thus utilizing triglyceride stores [31]. Dairy protein may also improve metabolic health by promoting changes in body composition in favor of increased lean body mass and decreased fat mass [32,33]. To improve the health status of those with higher carbohydrate intake, substituting carbohydrate-rich foods with proteins and fats from healthy sources may make it easier to dramatically change their diet [21,34].

### 4.4. Metabolic Syndrome and Inflammation

Metabolic syndrome is a proinflammatory state characterized by increased CRP levels [4]. In the present study, we observed a stronger association between carbohydrate intake and metabolic syndrome among those with low levels of CRP. This association was also observed for central obesity and dyslipidemia. Carbohydrates and other dietary components in the high-carbohydrate group may affect proinflammatory status and the level of CRP [35,36]. A higher level of CRP is related to an increased risk of metabolic syndrome and its components, which are associated with underlying inflammatory processes [3,4]. CRP is produced in the liver, primarily in response to interleukin-6, tumor necrosis factor-α and interleukin-1, each of which has been implicated in insulin-resistance pathways. A higher carbohydrate diet may induce higher rates of interleukin-6 secretion from adipocytes and thus increase the level of CRP [36]. Our findings show that the role of diet could be more significant among those with a low inflammatory status, implying the importance of prevention in managing metabolic syndrome. 

### 4.5. Study Limitation

This study had several limitations that should be considered. First, our data are based on a cross-sectional study; thus, it is difficult to explain the causal relationship between carbohydrate intake and metabolic syndrome. Second, dietary information was obtained from a self-reported FFQ; thus, measurement errors in dietary assessment are inevitable. Because underreporting of energy intake is a major source of bias in dietary surveys, we conducted energy adjustment to mitigate the effects of measurement error in data collected via self-reported dietary assessment instruments [20]. Third, carbohydrate intake is very high in this population. Therefore, variation in the intake of carbohydrates (typically providing 60% to 70% of daily energy) is smaller than that for other nutrients. Fourth, our data did not provide the information of menopausal status. However, our analyses were adjusted by age, so it could mitigate the lack of this information. Finally, this study included only Korean women. Thus, further studies are required to examine the association between carbohydrate intake and metabolic syndrome according to race and gender, and the underlying mechanism. 

## 5. Conclusions

The present study found that high carbohydrate intake was associated with an increased prevalence of metabolic syndrome and abdominal obesity. This association could differ according to individuals’ inflammatory status and other food choices. Socioeconomic factors affect carbohydrate intake, and a higher proportion of carbohydrate intake results in an imbalanced diet with poor diet quality. These findings may help to develop more specialized strategies to prevent metabolic syndrome in vulnerable social groups. Further investigations are needed to determine an appropriate carbohydrate intake level and diet composition. The implementation of dietary interventions, such as providing food vouchers, could be one of the effective strategies to allow those with low socioeconomic status to achieve a balanced diet with variety.

## Figures and Tables

**Table 1:** Association of carbohydrate intake with the risk of metabolic syndrome and its components.

Carbohydrate Intake ^1^	No. (%)		Crude OR (95% CI)	Adjusted OR (95% CI) ^2^
	Controls	Cases		
Metabolic syndrome				
Q1	768 (25.0)	200 (16.4)	1.0 (ref)	1.0 (ref)
Q2	768 (25.0)	225 (18.4)	1.13 (0.91–1.40)	0.99 (0.79–1.25)
Q3	768 (25.0)	278 (22.8)	1.39 (1.13–1.71)	1.00 (0.80–1.25)
Q4	769 (25.0)	518 (42.4)	2.59 (2.14–3.13)	1.34 (1.08–1.66)
*p* for trend			<0.001	0.004
Elevated waist circumference				
Q1	718 (25.9)	250 (16.4)	1.0 (ref)	1.0 (ref)
Q2	717 (25.9)	276 (18.1)	1.11 (0.91–1.35)	1.04 (0.84–1.28)
Q3	697 (25.1)	349 (22.9)	1.44 (1.19–1.74)	1.02 (0.83–1.26)
Q4	640 (23.1)	647 (42.5)	2.90 (2.42–3.48)	1.32 (1.08–1.62)
*p* for trend			<0.001	0.007
High blood pressure				
Q1	637 (26.4)	331 (17.6)	1.0 (ref)	1.0 (ref)
Q2	618 (25.6)	375 (19.9)	2.17 (0.97–1.41)	0.99 (0.81–1.21)
Q3	594 (24.6)	452 (24.0)	1.46 (1.22–1.75)	0.98 (0.80–1.19)
Q4	564 (23.4)	723 (38.4)	2.47 (2.08–2.93)	1.07 (0.87–1.31)
*p* for trend			<0.001	0.623
High triglycerides				
Q1	721 (23.9)	247 (19.4)	1.0 (ref)	1.0 (ref)
Q2	719 (23.8)	274 (21.5)	1.11 (0.91–1.36)	1.01 (0.82–1.23)
Q3	755 (25.0)	291 (22.9)	1.13 (0.92–1.37)	0.95 (0.78–1.17)
Q4	826 (27.3)	461 (36.2)	1.63 (1.36–1.96)	1.19 (0.97–1.47)
*p* for trend			<0.001	0.118
High fasting blood glucose				
Q1	861 (23.0)	107 (19.4)	1.0 (ref)	1.0 (ref)
Q2	870 (23.3)	123 (22.3)	1.14 (0.86–1.50)	1.03 (0.78–1.37)
Q3	905 (24.2)	141 (25.5)	1.25 (0.96–1.64)	1.08 (0.82–1.42)
Q4	1106 (29.6)	181 (32.8)	1.32 (1.02–1.70)	1.08 (0.82–1.44)
*p* for trend			0.027	0.508
Low HDL cholesterol				
Q1	536 (24.9)	432 (20.2)	1.0 (ref)	1.0 (ref)
Q2	512 (23.8)	481 (22.5)	1.17 (0.98–1.39)	1.11 (0.93–1.33)
Q3	511 (23.7)	535 (25.0)	1.30 (1.09–1.55)	1.18 (0.99–1.42)
Q4	595 (27.6)	692 (32.3)	1.44 (1.22–1.71)	1.19 (0.99–1.43)
*p* for trend			<0.001	0.040

Abbreviations: CI, confidence interval; HDL, high-density lipoprotein; OR, odds ratio; ref, reference; Q, quartile. ^1^ The intake levels of carbohydrates were categorized into quartiles according to the distribution of the control group (Q1: < 67.0, Q2: 67.0–71.4, Q3: 71.4–75.2, Q4: ≥ 75.2% of energy). ^2^ Tests of association were from logistic regression and were adjusted for age, residence area, and education.

**Table 2:** General characteristics of the study participants according to the quartiles of carbohydrate intake in controls ^1^.

	Quartiles of Carbohydrate Intake ^3^				
	Q1 (*n* = 768)	Q2 (*n* = 768)	Q3 (*n* = 768)	Q4 (*n* = 769)	*p*-Value ^4^
Age (years)					
<50	546 (71.1)	454 (59.1)	414 (53.9)	263 (34.2)	<0.001
50–60	147 (19.1)	186 (24.2)	186 (24.2)	235 (30.6)	
≥60	75 (9.8)	128 (16.7)	168 (21.9)	271 (35.2)	
BMI (kg/m^2^)					
<23	300 (39.1)	229 (33.7)	262 (34.1)	301 (39.1)	0.063
23–25	227 (29.6)	238 (31.0)	221 (28.8)	206 (26.8)	
≥25	241 (31.4)	271 (35.3)	285 (37.1)	262 (34.1)	
Residential location					
Rural area	230 (30.0)	201 (26.2)	318 (41.4)	558 (72.6)	<0.001
Urban area	538 (70.1)	567 (73.8)	450 (58.6)	211 (27.4)	
Marital status					
No	69 (9.0)	102 (13.1)	100 (13.0)	120 (15.6)	0.001
Yes	699 (91.0)	664 (86.7)	664 (86.9)	644 (84.3)	
Unknown	0 (0)	2 (0.3)	4 (0.5)	5 (0.7)	
Occupation					
Professional, Office	29 (3.8)	26 (3.4)	12 (1.7)	13 (1.7)	<0.001
Service, Sales	114 (14.8)	81 (10.5)	78 (10.2)	56 (7.3)	
Agriculture, manufacturing	107 (14.0)	109 (14.2)	175 (22.8)	309 (40.2)	
Housewife, others	517 (67.3)	552 (71.9)	501 (65.2)	385 (50.1)	
Unknown	1 (0.1)	0 (0)	2 (0.3)	3 (0.8)	
Education level					
Middle school or less	343 (44.7)	406 (52.9)	501 (65.2)	629 (81.8)	<0.001
High school	345 (44.9)	289 (37.6)	212 (27.6)	110 (14.3)	
College or more	78 (10.2)	68 (8.9)	49 (6.4)	24 (3.1)	
Unknown	0 (0.3)	5 (0.7)	6 (0.8)	6 (0.8)	
Household income ^2^					
Low (<200 won)	385 (50.1)	429 (55.9)	512 (66.7)	625 (81.4)	<0.001
Medium (200–400 won)	296 (38.5)	260 (33.9)	196 (25.5)	104 (13.5)	
High (≥400 won)	77 (10.0)	64 (8.3)	46 (6.0)	15 (2.0)	
Unknown	10 (1.3)	15 (2.0)	14 (1.8)	25 (3.3)	
Alcohol consumption					
Never	458 (59.6)	541 (70.4)	521 (67.8)	574 (74.6)	<0.001
Ever	309 (40.3)	223 (29.2)	245 (32.0)	189 (24.8)	
Unknown	1 (0.1)	4 (0.5)	2 (0.3)	6 (0.8)	
Smoking status					
Never	712 (92.7)	731 (95.2)	731 (95.2)	722 (93.9)	0.335
Ever	41 (5.4)	29 (3.8)	33 (4.3)	28 (3.7)	
Unknown	15 (2.0)	8 (1.0)	4 (0.5)	19 (2.5)	
Physical activity (METs/day)					
Q1 (<720)	191 (24.9)	193 (25.1)	169 (22.0)	204 (26.5)	<0.001
Q2 (720–1155)	223 (29.0)	243 (31.6)	206 (26.8)	134 (17.4)	
Q3 (1155–1965)	250 (32.6)	214 (27.9)	187 (24.3)	133 (17.3)	
Q4 (≥1965)	87 (11.3)	101 (13.2)	189 (24.6)	262 (34.1)	
Unknown	17 (2.2)	17 (2.2)	17 (2.2)	36 (4.7)	

Abbreviations: BMI, body mass index; METs, metabolic equivalents. ^1^ Data are presented as *n* (%). ^2^ Unit is 10,000 won in Korean currency ($1 = 1103.50 Korean won as of 24 December 2020). ^3^ Subjects were divided into four groups based on carbohydrate intake among those having no metabolic syndrome (Q1: < 67.0, Q2: 67.0–71.4, Q3: 71.4–75.2, Q4: ≥ 75.2% of energy). ^4^ Tests of association by chi-square test (categorical variables).

**Table 3:** Association of carbohydrate intake with the risk of metabolic syndrome and its components, stratified by CRP level.

Carbohydrate Intake ^1^	Low CRP ^2^		High CRP	
	No. Controls/Cases	OR (95% CI) ^3^	No. Controls/Cases	OR (95% CI) ^3^
Metabolic syndrome				
Q1	341/43	1.0 (ref)	427/157	1.0 (ref)
Q2	298/55	1.40 (0.90–2.20)	470/170	0.86 (0.66–1.12)
Q3	295/78	1.65 (1.08–2.52)	473/200	0.82 (0.63–1.07)
Q4	290/132	1.84 (1.21–2.80)	479/386	1.17 (0.91–1.51)
*p* for trend		0.003		0.147
Elevated waist circumference				
Q1	308/76	1.0 (ref)	410/174	1.0 (ref)
Q2	276/77	1.09 (0.76–1.58)	441/199	0.99 (0.76–1.28)
Q3	278/95	1.12 (0.78–1.61)	419/254	0.95 (0.73–1.23)
Q4	234/188	1.76 (1.24–2.51)	406/459	1.13 (0.87–1.45)
*p* for trend		0.002		0.344
High blood pressure				
Q1	284/100	1.0 (ref)	353/231	1.0 (ref)
Q2	249/104	1.06 (0.74–1.50)	369/271	0.95 (0.74–1.21)
Q3	242/131	1.06 (0.75–1.49)	352/321	0.94 (0.74–1.21)
Q4	208/214	1.16 (0.82–1.64)	356/509	1.01 (0.79–1.30)
*p* for trend		0.464		0.972
High triglyceride				
Q1	325/59	1.0 (ref)	396/188	1.0 (ref)
Q2	282/71	1.29 (0.87–1.90)	437/203	0.89 (0.70–1.14)
Q3	286/87	1.49 (1.02–2.18)	469/204	0.78 (0.61–1.00)
Q4	311/111	1.53 (1.03–2.26)	515/350	1.06 (0.83–1.35)
*p* for trend		0.026		0.729
High fasting blood glucose				
Q1	359/25	1.0 (ref)	502/82	1.0 (ref)
Q2	324/29	1.15 (0.65–2.02)	546/94	0.98 (0.71–1.36)
Q3	334/39	1.37 (0.80–2.35)	571/102	0.98 (0.71–1.35)
Q4	371/51	1.45 (0.84–2.51)	735/130	0.97 (0.70–1.34)
*p* for trend		0.165		0.892
Low HDL cholesterol				
Q1	248/136	1.0 (ref)	288/296	1.0 (ref)
Q2	190/163	1.51 (1.12–2.03)	322/318	0.91 (0.73–1.14)
Q3	183/190	1.80 (1.34–2.42)	328/345	0.92 (0.73–1.15)
Q4	213/209	1.57 (1.15–2.14)	382/483	1.00 (0.79–1.26)
*p* for trend		0.001		0.976

Abbreviations: CI, confidence interval; CRP, c-reactive protein; HDL, low-density lipoprotein; OR, odds ratio; ref, reference; Q, quartile. ^1^ The intake levels of carbohydrates were categorized into quartiles according to the distribution of the control group (Q1: < 67.0, Q2: 67.0–71.4, Q3: 71.4–75.2, Q4: ≥ 75.2% of energy). ^2^ The level of CRP was categorized into two groups (cutoff: 1 mg/dL). ^3^ Tests of association were from logistic regression and were adjusted for age, residence area, and education.

**Table 4:** Association between dairy food intake and the risk of metabolic syndrome and its components, stratified by the level of carbohydrate intake ^1^.

	Low Carbohydrate Intake			High Carbohydrate Intake		
Dairy Food Intake	Low	High	*p*-Value	Low	High	*p*-Value
Metabolic syndrome						
No. controls/cases	563/168	973/257	0.333	973/571	564/225	0.014
OR (95% CI)^2^	1.0 (ref)	0.89 (0.71–1.13)		1.0 (ref)	0.78 (0.64–0.95)	
Elevated waist circumference						
No. controls/cases	510/221	925/305	0.062	818/726	519/270	<0.001
OR (95% CI) ^2^	1.0 (ref)	0.81 (0.65–1.01)		1.0 (ref)	0.72 (0.59–0.87)	
High blood pressure						
No. controls/cases	461/270	794/436	0.444	732/812	426/363	0.264
OR (95% CI) ^2^	1.0 (ref)	0.92 (0.75–1.14)		1.0 (ref)	0.90 (0.74–1.09)	
High triglycerides						
No. controls/cases	531/200	909/321	0.431	1006/538	575/214	0.001
OR (95% CI) ^2^	1.0 (ref)	0.92 (0.74–1.14)		1.0 (ref)	0.72 (0.59–0.87)	
High fasting glucose						
No. controls/cases	634/97	1097/133	0.051	1335/209	676/113	0.601
OR (95% CI) ^2^	1.0 (ref)	0.75 (0.57–1.00)		1.0 (ref)	1.07 (0.83–1.38)	
Low HDL cholesterol						
No. controls/cases	384/347	664/566	0.624	701/843	405/384	0.015
OR (95% CI) ^2^	1.0 (ref)	0.95 (0.79–1.15)		1.0 (ref)	0.80 (0.67–0.96)	

Abbreviations: CI, confidence interval; HDL, high-density lipoprotein; OR, odds ratio. ^1^ The intake levels of carbohydrate and dairy food were categorized into two groups according to the distribution in the control group. ^2^ Tests of association were from logistic regression and were adjusted for age, residence area, and education.

## Supplementary Materials

The following are available online at https://www.mdpi.com/article/10.3390/nu13093098/s1. Table S1: General characteristics of the study participants according to metabolic syndrome status. Table S2: Daily intake of energy and nutrients in the subjects according to carbohydrate intake in controls. Table S3: Food-group intake according to the level of carbohydrate intake in controls. Table S4: Association between carbohydrate intake and the risk of metabolic syndrome, stratified by the intakes of different food groups.
