# Supplementary material for: Association between Carbohydrate Intake and the Prevalence of Metabolic Syndrome in Korean Women
Source: Nutrients. 2021 Sep 3;13(9):3098. doi: 10.3390/nu13093098 (PMC8465012; doi:10.3390/nu13093098)
Supplement: Supplementary file 1 [file nutrients-13-03098-s001.zip › nutrients-1350105-supplementary.pdf]

Supplementary Tables

Table S1. General characteristics of the study participants according to metabolic syndrome status[1]

	Controls (n=3073)	Cases (n=1221)	*p*-value[3]
Age (years)			
<50	1677 (54.5)	302 (24.7)	<0.001
50-60	754 (24.5)	386 (31.6)	
≥60	642 (20.9)	533 (43.7)	
BMI (kg/m²)			
<23	1122 (36.5)	110 (9.0)	<0.001
23-25	892 (29.0)	229 (18.8)	
≥25	1059 (34.5)	882 (72.2)	
Residential location			
Rural area	1307 (42.5)	776 (63.6)	<0.001
Urban area	1766 (57.5)	445 (36.5)	
Marital status			
No	391 (12.7)	241(19.7)	<0.001
Yes	2671 (87.2)	972 (80.1)	
Unknown	11 (0.4)	8 (0.7)	
Occupation			
Professional, Office	80 (2.6)	15 (1.2)	<0.001
Service, Sales	329 (10.7)	81 (6.6)	
Agriculture, manufacturing	700 (22.8)	385 (31.5)	
Housewife, others	1955 (63.6)	736 (60.3)	
Unknown	9 (0.3)	4 (0.3)	
Education level			
Middle school or less	1879 (61.1)	990 (81.1)	<0.001
High school	956 (31.1)	175 (14.3)	
College or more	219 (7.1)	44 (3.6)	
Unknown	19 (0.6)	12 (1.0)	
Household income[2]			
Low (<200 won)	1951 (63.5)	945 (77.4)	<0.001
Medium (200-400 won)	856 (27.9)	207 (17.0)	
High (≥400 won)	202 (6.6)	48 (3.9)	
Unknown	64 (2.1)	21 (1.7)	
Alcohol consumption			
Never	2094 (68.1)	943(77.2)	<0.001
Ever	966 (31.6)	271 (22.3)	
Unknown	13 (0.4)	7 (0.6)	
Smoking status			
Never	2896 (94.2)	1133 (92.9)	0.018
Ever	131 (4.3)	73 (6.1)	
Unknown	46 (1.5)	15 (1.2)	
Physical activity (METs/d)			
Q1 (<720)	757 (24.6)	295 (24.2)	<0.001
Q2 (720-1155)	806 (26.2)	280 (22.9)	
Q3 (1155-1965)	784 (25.5)	253 (20.7)	
Q4 (≥1965)	639 (20.8)	358 (39.3)	
Unknown	87 (2.8)	35 (2.9)	

Abbreviations: BMI, body mass index; METs, metabolic equivalents

[1]Data are presented as n (%); [2]Unit is 10,000 won in Korean currency ($1 = 1,103.50 Korean won as of 24 December 2020); [3]Tests of association by chi-square test (categorical variables).

Table S2. Daily intake of energy and nutrients in the subjects according to carbohydrate intake in controls[1]

	Quartiles of Carbohydrate Intake[2]				*p*-value[3]
	Q1 (n = 768)	Q2 (n = 768)	Q3 (n =768)	Q4 (n = 769)	
Energy (kcal/day)	1900.4 ± 691.3	1854.8 ± 494.2	1786.3 ± 502.4	1935.4 ± 745.7	<0.001
Macronutrients					
Carbohydrate (g/day)	311.6 ± 22.2	346.7 ± 6.4	366.4 ± 5.5	393.3 ± 13.2	<0.001
Protein (d/day)	80.7 ± 10.3	70.6 ± 6.4	64.2 ± 5.5	56.2 ± 6.2	<0.001
Fat (g/day)	47.6 ± 9.8	34.7 ± 3.7	27.8 ± 3.4	19.2 ± 4.7	<0.001
%Energy from carbo-hydrate	62.9 ± 4.7	70.0 ± 2.1	74.2 ± 2.0	79.3 ± 2.8	<0.001
%Energy from protein	16.0 ± 2.0	14.0 ± 1.3	12.7 ± 1.2	11.2 ± 1.3	<0.001
%Energy from fat	20.5 ± 4.0	15.1 ± 2.2	12.0 ± 2.1	8.4 ± 2.5	<0.001
Minerals					
Calcium (mg)	632.5 ± 237.3	558.0 ± 182.9	478.8 ± 165.9	390.6 ± 155.0	<0.001
Phosphorus (mg)	1205.7 ± 183.5	1116.6 ± 143.2	1033.2 ± 132.2	928.4 ± 18.6	<0.001
Iron (mg)	12.8 ± 3.0	11.8 ± 3.0	11.0 ± 2.7	10.2 ± 2.9	<0.001
Potassium (mg)	2959.6 ± 785.3	2723.7 ± 652.9	2552.2 ± 661.3	2464.1 ± 816.8	<0.001
Sodium (mg)	3321.8 ± 1371.0	3222.6 ± 1195.4	3058.1 ± 1286.9	3094.7 ± 1485.8	<0.001
Zinc (ug)	10.0 ± 2.8	9.1 ± 1.6	8.6 ± 1.6	7.9 ± 1.3	<0.001
Vitamins					
Vitamin A (RE)	646.0 ± 357.4	564.3 ± 302.1	505.3 ± 290.9	484.7 ± 350.3	<0.001
Vitamin B1 (mg)	1.5 ± 0.3	1.3 ± 0.2	1.2 ± 0.2	1.1 ± 0.2	<0.001
Vitamin B2 (mg)	1.3 ± 0.3	1.1 ± 0.2	1.0 ± 0.2	0.8 ± 0.2	<0.001
Niacin (mg)	18.7 ± 3.3	16.2 ± 2.4	14.9 ± 2.0	13.9 ± 2.1	<0.001
Vitamin B6 (mg)	2.0 ± 0.4	1.9 ± 0.3	1.8 ± 0.3	1.8 ± 0.4	<0.001
Vitamin C (mg)	140.1 ± 71.5	130.5 ± 683	128.7± 64.2	154.7 ± 98.7	<0.001
Vitamin E (mg)	11.4 ± 3.5	9.8 ±2.6	9.2 ± 2.5	8.7 ± 3.0	<0.001
Folate (ug)	276.5 ± 93.7	266.6 ± 84.9	250.5 ± 79.8	249.6 ± 98.3	<0.001

[1]Data are expressed as the mean ± standard deviation; [2]The intake levels of carbohydrates were categorized into quartiles according to the distribution in the control group (Q1: < 67.0, Q2: 67.0-71.4, Q3: 71.4-75.2, Q4: ≥ 75.2 % of energy); [3]General linear model (GLM) was used to analyze the p-value after adjusting for age, residential location, and education

Table S3. Food group intake according to the level of carbohydrate intake in controls[1]

Food group (g/day)	Quartiles of carbohydrate intake[2]				*p*-value[3]
	Q1 (n = 768)	Q2 (n = 768)	Q3 (n = 768)	Q4 (n = 769)	
Grains	508.0 ± 149.8	649.4 ± 111.1	722.8 ± 108.5	822.5 ± 173.7	<0.001
Noodles	23.6 ± 22.3	20.1 ± 18.6	29.7 ± 20.4	15.0 ± 17.8	<0.001
Rice cake	6.9 ± 13.5	5.6 ± 8.8	6.0 ± 11.5	5.7 ± 14.0	0.007
Breads	19.7 ± 26.1	14.8 ± 20.8	12.0 ± 19.3	7.2 ± 13.2	<0.001
Snacks	14.5 ± 19.8	10.3 ± 14.2	8.0 ± 12.1	5.1 ± 9.5	<0.001
Potatoes	25.2 ± 25.7	24.6 ± 24.6	25.5 ± 33.8	23.5 ± 35.1	0.222
Legumes	50.8 ± 49.8	45.7 ± 44.6	41.8 ± 43.5	31.0 ± 28.6	<0.001
Nuts	5.1 ± 8.2	3.9 ± 7.4	3.4 ± 9.5	3.1 ± 7.2	<0.001
Kimchi	172.5 ± 125.2	191.0 ± 117.8	194.5 ± 129.1	221.3 ± 156.2	<0.001
Green, yellow vegetables	116.3 ± 99.8	95.0 ± 75.2	91.0 ± 81.5	95.3 ± 95.6	<0.001
Light-colored vegetables	47.8 ± 38.8	41.4 ± 41.5	36.6 ± 30.1	33.1 ± 35.0	<0.001
Mushrooms	12.9 ± 13.7	10.1 ± 14.9	7.5 ± 10.7	4.7 ± 7.7	<0.001
Fruits/fruit juice	275.0 ± 233.7	263.5 ± 224.9	276.6 ± 216.6	399.4 ± 370.6	<0.001
Chickens	13.4 ± 16.3	7.7 ± 8.9	5.3 ± 6.3	3.6 ± 5.1	<0.001
Read meats	77.8 ± 51.1	42.9 ± 24.7	29.4 ± 19.8	17.1 ± 13.7	<0.001
Processed meat	2.2 ± 5.0	1.3 ± 3.4	0.8 ± 2.7	0.3 ± 1.4	<0.001
Eggs	19.2 ± 18.9	14.9 ± 16.2	11.5 ± 13.7	7.5 ± 10.5	<0.001
Fish	45.7 ± 36.6	33.1 ± 24.7	24.2 ± 18.4	14.7 ± 13.8	<0.001
Salted seafood	2.8 ± 6.1	1.7 ± 3.3	1.9 ± 5.2	1.4 ± 4.5	<0.001
Seaweeds	2.9 ± 2.6	2.4 ± 2.0	2.1 ± 2.0	1.8 ± 1.8	<0.001
Milk	127.7 ± 142.7	105.1 ± 114.3	76.7 ± 100.0	36.3 ± 67.1	<0.001
Dairy products	53.1 ± 64.3	50.7 ± 64.3	42.7 ± 59.9	29.3 ± 60.2	<0.001

[1]Data are expressed as the mean ± standard deviation; [2]The intake levels of carbohydrates were categorized into quartiles according to the distribution in the control group (Q1: < 67.0, Q2: 67.0-71.4, Q3: 71.4-75.2, Q4: ≥ 75.2 % of energy); [3]General linear model (GLM) was used to analyze the p-value after adjusting for age, residential location, and education

Table S4. Association between carbohydrate intake and the risk of metabolic syndrome, stratified by the intakes of different food groups[1]

Food Group	All Women			Low Carbohydrate Intake			High Carbohydrate Intake		
	Low	High	*p*-value	Low	High	*p*-value	Low	High	*p*-value
Kimchi									
No. Controls/cases	1536/594	1537/627	0.117	817/219	719/206	0.523	719/375	818/421	0.119
OR (95% CI)[2]	1.0 (ref)	0.89 (0.78–1.03)		1.0 (ref)	0.93 (0.74–1.17)		1.0 (ref)	0.87 (0.72–1.04)	
Vegetables without kimchi									
No. Controls/cases	1536/619	1537/602	0.190	660/171	876/254	0.456	876/448	661/348	0.148
OR (95% CI)[2]	1.0 (ref)	1.10 (0.95–1.27)		1.0 (ref)	1.09 (0.87–1.37)		1.0 (ref)	1.14 (0.95–1.37)	
Vegetables									
No. Controls/cases	1537/576	1536/645	0.602	744/191	792/234	0.784	793/385	744/411	0.521
OR (95% CI)[2]	1.0 (ref)	1.04 (0.90–1.20)		1.0 (ref)	1.03 (0.82–1.30)		1.0 (ref)	1.06 (0.89–1.27)	
Fruits									
No. Controls/cases	1537/594	1536/627	0.751	834/232	702/193	0.735	703/362	834/434	0.603
OR (95% CI)[2]	1.0 (ref)	1.02 (0.89–1.18)		1.0 (ref)	0.96 (0.76–1.21)		1.0 (ref)	1.05 (0.88–1.26)	
Dairy foods									
No. Controls/cases	1536/739	1537/482	0.004	563/168	973/257	0.333	973/571	564/225	0.014
OR (95% CI)[2]	1.0 (ref)	0.81 (0.70–0.93)		1.0 (ref)	0.89 (0.71–1.13)		1.0 (ref)	0.78 (0.64–0.95)	
Meats									
No. Controls/cases	1537/774	1536/447	0.087	294/100	1242/325	0.286	1243/674	294/122	0.896
OR (95% CI)[2]	1.0 (ref)	0.88 (0.75–1.02)		1.0 (ref)	0.86 (0.66–1.13)		1.0 (ref)	1.02 (0.79–1.30)	

Abbreviations: CI, confidence interval; OR, odds ratio

[1]The intake levels of carbohydrates and other food groups were categorized into two groups according to the distribution in the control group.

[2]Tests of association were from logistic regression and were adjusted for age, residence area and education
